# Increasing unemployment rate amongst health professionals: Will there be jobs for newly graduated South African audiologists post-COVID-19?

**DOI:** 10.4102/sajcd.v69i2.909

**Published:** 2022-08-01

**Authors:** Vera-Genevey Hlayisi

**Affiliations:** 1 Division of Communication Sciences and Disorders, Department of Health and Rehabilitation Sciences, Faculty of Health Sciences, University of Cape Town, Cape Town, South Africa

**Keywords:** employment, unemployment, human-resources, healthcare, audiology

## Abstract

**Background:**

Before the coronavirus disease 2019 (COVID-19) pandemic in early 2020, the unemployment rate in South Africa was at its highest in history at 29.1%. During the COVID-19 pandemic to date, unemployment rose even higher to 35.3%. In this context, there has been an increase in the number of unemployed health professionals in South Africa.

**Objectives:**

This study aimed to determine the employment rates of newly graduated South African audiologists and identify the challenges in obtaining and maintaining employment for audiologists in South Africa.

**Methods:**

A descriptive online survey design was used. Participants were recruited online through professional association webpages using the snowball sampling technique. All qualified audiologists registered with the Health Professionals Council of South Africa were eligible to participate.

**Results:**

A total of 132 audiologists completed the survey. In the first-year postgraduation, 16% of the participants were unemployed, and this increased to 19% in the second-year postgraduation. In the majority (81%) of employed participants, almost a fifth (19%) were working within non-audiology/healthcare fields. The most common workplace challenges reported were remuneration (37%) followed by lack of resources (18%), workload (18%), work environment (10%), working hours (9%) and, lastly, interprofessional relationships (8%).

**Conclusion:**

Findings from this study are the first to document employment rates amongst South African audiologists. These findings have the potential to influence the critical discourse on hearing healthcare human resource planning, hearing healthcare labour capacity and potential for growth in the South African context post-COVID-19.

## Introduction

Unemployment is a socio-political challenge worldwide and a major challenge in South Africa where the current national unemployment rate is the highest (Du Toit, De Witte, Rothmann, & Van den Broeck, 2018; Statistics South Africa, 2021). Statistics South Africa (2022) reports a 35.3% official unemployment rate, the highest since 2008, which translates to almost 8 million people who are able (skilled) to work and actively seeking jobs (Alenda-Demoutiez & Mügge 2019). The expanded unemployment rate, which would include those who are not actively seeking jobs skilled (Alenda-Demoutiez & Mügge 2019) or otherwise, is much higher and is up to 46.2% of the South African population (Statistics South Africa, 2021). Even more staggering is the unemployment rate amongst the youth, which is currently at 65.5%.

More than 15 years ago, the growing unemployment crisis in South Africa was described then, as a ‘beast’, with an ‘effect on economic welfare, erosion of human capital, crime and social instability’ (Kingdon & Knight, 2007). In response to the annual increase in unemployment, the government has shown a wide range of policy efforts and interventions to curb the crisis and impact, especially amongst the youth (Du Toit et al., 2018; Lannoy, Graham, Patel, & Liebbrandt, 2018). One of the key interventions has been increasing access and quality of education with the aim of increasing the number of skilled youth that would be more employable as per labour market skills demand or need (De Witte, Rothmann, & Jackson, 2012; du Toit et al., 2018). In post-apartheid South Africa, increasing access, quality and levels of education have been a key focus as research has shown that educated or skilled young people stand a better chance of finding employment, faster than their peers with lower levels of schooling (Acquah, 2009; Lannoy et al., 2018; Mlatsheni & Rospabe, 2002). However, there seems to still be a persistent case of unemployment even amongst skilled graduate professionals in South Africa to date, despite vast efforts invested in access and quality of education (Lannoy et al., 2018). Graduate unemployment is problematic because it wastes scarce human capital that is detrimental to the economy in the long run (Oluwajodu, Blaauw, Greyling, Kleynhans, 2015).

Currently, South Africa’s labour market consists of formal and informal employment. Formal employment is defined as employment created by businesses or the government where an employee is hired under established working agreements (Alenda-Demoutiez & Mügge, 2019; Quain, 2018). In 2014, only 25% of South Africa’s workforce were skilled workers with formal employment, that is, including graduate health workers (Statistics South Africa, 2014). In 2016, 405 000 South African citizens with tertiary-level education including health professionals were unemployed (Mngoma, 2016). Graduate unemployment is an important area of study because the South African economy experiences severe skills shortages, however, simultaneously unable to generate and sustain sufficient job opportunities for skilled labour (Pauw, Bhorat, & Goga, 2006). The issue of graduate unemployment, therefore, contradicts studies suggesting that the higher a participant’s education level, the higher the probability of finding employment; for example, health professional graduates with specialised education are expected to be fully absorbed into the skills starved labour market (Cohen & Moodley, 2012; Mncayi & Dunga, 2016; Oosthuizen, 2007). The South African healthcare economy and setting are complex (Coovardia, Jewkes, Barron, Sander, & McIntyre, 2009). There is a rise in health human resource and service wastage through unemployment, low worker retention and low overall system efficiency as influenced by the macro socio-economic and political issues that are not unique to South Africa but are in most developing African countries (Dovlo, 2005). The term *wastage* of health human resources is used by previous researchers in the field (Dovlo, 2005) to describe the loss in utility of health workers/health professionals resulting from underuse or non-use of trained personnel, unemployment, retrenchment or the labour force’s inability to absorb skilled graduates. Some researchers argue that many of the South African healthcare challenges are rooted in distinct features of the apartheid history, which sustained health inequity through its successive segregationist policies (Delobelle, 2013; Pillay, Tiwari, Kathard, & Chikte, 2020). To date, there has been a recent increase in unemployed health professionals, including audiologists (Maqhina, 2019; Msomi, 2019; Statistics South Africa, 2021; Van Dyk, 2017).

Audiologists are the primary hearing healthcare professionals involved in the identification, prevention and evaluation of auditory and balance disorders. Moreover, audiologists are the single most important resources for the rehabilitation of hearing loss (Gelfand, 2009). Audiology is relatively young amongst other South African health professions and has been growing in the last half-century from an adjunct to Speech Therapy to its own autonomous profession (Swanepoel, 2006). Currently, audiology training as a singular, autonomous profession is available at some of the seven higher education institutions offering the degree in South Africa with approximately 100–150 new graduates recorded annually (Department of Higher Education [DHET], 2017; Singh et al., 2015; Swanepoel, 2006). It is worth noting that this is a low number of audiologists entering into healthcare system to meet the high need of hearing health services as influenced by the disease and disability burden in South Africa (Khoza-Shangase & Mophosho, 2018; Pillay et al., 2020). Lower supplies of rehabilitation health professionals like audiologists is a common challenge in low- and middle-income countries, including many located in sub-Saharan Africa, where the need of the skills tends to be greatest (Gupta, Castillo-Laborde, & Landry, 2011; Pillay et al., 2020). The Health Professions Council of South Africa (2022) iregister indicates approximately 1175 audiologists and 2205 speech therapists and audiologists currently registered to provide hearing healthcare services in the country of over 60 million in population size. Pillay et al. (2020) estimated a ratio of 39 audiologists per 1 million population, based on the 2017 World Bank estimate for the population of South Africa of 56.7 million. Audiologists in South Africa are mostly employed and function in the public healthcare system (Khoza-Shangase & Mophosho, 2018; Pillay et al., 2020; Swanepoel, 2006). The South African public healthcare system is unfortunately marred with several challenges including well-documented lack of skilled professionals, infrastructural constraints, limited well-functioning facilities, general lack of resources for the size of the population, risk versus benefit assessment predicaments and challenges with translating policies into practice (Barratt, Khoza-Shangase, & Msimang, 2012; Khoza-Shangase, Kanji, Petrocchi-Bartal, & Farr, 2017; Khoza-Shangase & Mophosho, 2018). These challenges affect mostly the already vulnerable parts of the population such as the poor with communication disabilities such as hearing loss (Khoza-Shangase et al., 2017; McKenzie, 1992; Wylie McAllister, Davidson, & Marshall, 2016).

It is also worth noting that the coronavirus disease 2019 (COVID-19) pandemic brought with it many challenges that will undoubtedly have an impact on unemployment or employment across many sectors, including audiology. Manchaiah, Eikelboom, Bennett and Swanepoel (2022) in their international survey with audiologists exploring the effects of COVID-19 on the workplace state that the pandemic has resulted in significant disruptions to audiological practice. Some of their key findings show that 97% of audiologists surveyed reported significant changes to their workplace, with up to 76.4% reporting reduced caseloads and 38.7% reported reduced working hours. COVID-19 will undeniably alter the practice of audiology, and emerging research is already showing an increase in the need to adapt and incorporate telehealth in audiology practice (Eikelboom et al., 2022; Manchaiah et al., 2022). Some of these early research reports on workload, work hours and incorporation of telehealth are likely to inform hearing healthcare human resource policies and planning, hearing healthcare labour market needs versus capacity and hearing healthcare context and future. Therefore, this study sought to investigate and describe challenges in obtaining and maintaining employment of audiologists as scarce skilled health human resources in the context of the rising unemployment rates in South Africa.

## Research methodology

### Research design

A descriptive cross-sectional online survey was used. A descriptive survey was chosen as both a research design and a tool for the data collection of this study (Barbour, 2014). The study design was chosen based on its many advantages including its cost-effective nature and its ability to overcome geographical boundaries when seeking to collect data from widely dispersed participants (Barbour, 2014).

### Study setting

This study was facilitated online using the web-based SurveyMonkey platform with target participants from the South African hearing healthcare sector that comprises both public and privately practicing audiologists. An online survey setting was chosen as it would enable target participants to have anonymity to share their responses to the research question openly and honestly (Barbour, 2014).

### Participants

#### Sample size

The sample size was calculated using an online calculator on the SurveyMonkey platform. An acceptable margin of error for survey studies is between 5% and 10% (Suresh & Chandrashekara, 2012). A lower margin of error increases the value of the sample, and the accuracy of the value reduces the risk of the sample not representing the population under investigation (Singh & Masuku, 2014). Using these parameters, 330 audiologists were calculated as the needed sample size. However, the response rate to previous South African research requiring audiologists to complete online surveys has been poor. In a recent South African study by Makhoba and Joseph (2016), only 45 responses were obtained from 1440 audiologists invited to complete the survey. Therefore, a sample size of 200 audiologists was targeted as the more feasible number to reach.

#### Sampling and recruitment method

The snowball sampling technique was used to allow for recruited participants to recruit other eligible participants they had access to (Etikan, Alkassim, & Abubakar, 2015). This method was appropriate based on the limited time and budget for this study. Employing this sampling method further increased the chances of having a diverse sample. Prospective participants were recruited online. Audiologists known by the researcher were sent a formal email to invite them to participate in the research study. The email contained an informational letter with all details of the study and the link to the survey. Before completing the survey, participants were required to provide electronic informed consent. Within the email, a request was made to forward the email to other audiologists they were in contact with. Additionally, audiologists were recruited on various social media platforms including Facebook and WhatsApp.

#### Inclusion criteria

For inclusion in the study, participants had to be graduate audiologists registered with the Health Professions Council of South Africa.

### Study materials

#### Survey tool

A 24-item questionnaire was designed for the study to collect data on (1) participant demographics, (2) education information and (3) unemployment or employment status and details. The questionnaire response formats included closed sets like multiple choices and open-ended narratives. The questionnaire was piloted prior study commenced to determine validity.

#### Face and content validity of data collection tool

According to previous research using questionnaires, face and content validity were essential psychometric properties to measure when developing such instruments (Connell et al., 2018). Face validity refers to whether the instrument appears to measure what it was intended to measure (Connell et al., 2018). Content validity refers to whether the instrument covers all aspects of content related to the construct it is being measured (Heale & Twycross, 2015). To ensure face and content validity, seven audiologists provided feedback on the (1) overall appearance (e.g. ease of response, response formats, etc.), (2) content covered and (3) overall flow in the design of the questionnaire. Feedback from the seven audiologists included changing some questions to be open ended to allow participants to give additional information, and in terms of the overall design, there was a need to redirect participants to the next appropriate question and sections of the survey. Based on this feedback, the questionnaire was modified prior the data collection commenced.

### Data collection

Data were collected online using the SurveyMonkey platform where the questionnaire was administered. The online link to the questionnaire was open for 6 months, and data were stored online on a password-protected cloud that only the researcher had access to.

### Data analysis

#### Quantitative data

Descriptive statistical methods were used to analyse quantitative data. Categorical data were analysed using frequency tables and percentages. Numerical data were analysed using means and standard deviation.

#### Qualitative data

A thematic analysis was conducted for the qualitative data collected. The following steps were included in the thematic analysis:

1. *Data familiarisation* – researcher read through the qualitative data collected and made notes to gain a better understanding of the data and to assist in a comprehensive analysis (Nowell, Norris, White, & Moules, 2017).
2. *Coding* – the data were then reviewed to determine key characteristics that could be coded. Codes were determined by highlighting similarities across participant responses.
3. *Categorisation* – Similar codes were grouped together in themes. All codes within the themes were then reviewed to establish whether they were classified into the appropriate themes.

### Ethical considerations

This study was guided by the ethical principles outlined by the World Medical Association (WMA) in the Declaration of Helsinki (World Medical Association, 2013). Ethical approval was granted by the Human Research and Ethics Committee (HREC) of the University of Cape Town (Reference no: 815/18).

## Results

A total of 219 audiologists responded to the study questionnaire, and 132 complete responses were collected. Only the results from the 132 completed questionnaires were included in the analysis.

### Participant demographics

Majority of the participants (89%) were female, South African youth between the ages of 25–34 (67%) (see Table 1 for more participant demographic data).

**TABLE 1:** Participant demographic information (*n* = 132).

Characteristics	*n*	%
**Sex**		
Female	118	89
Male	14	11
**Age range**		
18–24	13	10
25–34	88	67
35–44	27	20
45–54	2	2
> 55	2	1
**Level of qualification**		
Bachelor’s degree	103	78
Master’s degree	25	19
Doctoral degree	4	3
**South African citizen**		
Yes	130	99
No	2	1

### (Un)employment rate

#### First-year postgraduation

1. Of the 132 participants, the majority (84%) were employed by the government in the public sector through the community service (CS) program.
2. Of the 16% unemployed participants, 9% experienced a delay (1–2 months on average) in their employment through the CS program placement, 7% were not employed at all (not placed in CS program at all) and 1% did not meet requirements for CS program employment as they were non-SA citizens.

#### Second-year postgraduation

1. Of the 132 participants, the vast majority (81%) were employed. Important to note is that of the employed participants, up to 19% were working within non-audiology fields:
  - Employment characteristics of those working in audiology fields (*n* = 107) show that most participants were employed within the public sector (47%) and in clinical settings (52%) (see Table 2).
  - When asked to rate workplace challenges, most ratings (37%) were on remuneration followed by lack of resources (18%), workload (18%), work environment (10%), working hours (9%) and, lastly, interprofessional relationships (8%).

**TABLE 2:** Employment demographics within audiology (*n* = 107).

Characteristics	*n*	%
**Province**		
Eastern Cape	3	3
Free State	2	2
Gauteng	38	35
KwaZulu-Natal	10	9
Limpopo	16	15
Mpumalanga	7	6
Northern Cape	3	3
North West	3	3
Western Cape	25	23
**Sector**		
Public	50	47
Private	42	39
Both	15	14
**Employment contract**		
Full-time	81	76
Part-time	12	11
> 20 h per week	4	4
< 20 h per week	8	7
Other	2	2
**Area of practice: Participants ticked all that applied**		
Clinical	90	52
Teaching	31	18
Sales and training	17	10
Research	15	9
Other	19	11

The number of unemployed graduates increased to 19% in the second-year postgraduation (see Table 3 on the duration of unemployment and possible employment barriers). Challenges in obtaining employment are reported as themes in Table 4.

**TABLE 3:** Unemployed participants in the second-year postgraduation (*n* = 25).

Characteristics	*n*	%
**Duration of unemployment**		
< 3 months	15	60
< 6 months	6	24
1 year or more	4	16
**Reason for unemployment**		
Current postgraduate student	3	12
Pending community service placement	1	4
Limited job opportunities	24	26
HPCSA suspension	0	0

HPCSA, Health Professions Council of South Africa.

**TABLE 4:** Challenges obtaining employment.

Themes	Emerging codes
Limited job opportunities	Jobs far away from home
	Not enough jobs
	Not enough jobs in the province of choice
	Not enough jobs in the government
	Frozen job posts
	Post not funded
	Limited job advertisements
	No available locum posts
Discrimination	Private practice race issues
	Wearing head scarf not allowed
	Employer not wanting Indian employees
	Racial discrimination
Language barrier	Private practice wanting fluent Afrikaans speakers
	Afrikaans speaking audiologists required
Experience	Low remuneration for new graduates
	Don’t have enough experience for private
	Not enough years of experience
	Not enough experience for private sector
Qualification preference	Jobs for dually qualified audiologists only
	Only dually qualified posts available in Gauteng

## Discussion

### Unemployed health professionals

With the highest unemployment rate and the increasing challenges in healthcare human resourcing in South Africa, this study sought to describe challenges in obtaining and maintaining employment for audiologists (Pillay et al., 2020; Statistics South Africa, 2021). The main findings of this study indicate that up to 16% of audiologists are unemployed in their first-year postgraduation, and this increases to 19% in the second-year postgraduation. An unemployment rate between 16% and 19% is very high in the context of the estimated total annual number of audiology graduates from the seven institutions that offer the audiology program in South Africa, which is approximately between 100 and 150 graduates (DHET, 2017; Singh et al., 2015; Swanepoel, 2006), thus implying the possibility of an entire class of audiology graduates from one institution potentially unemployed in their first- and second-year postgraduation.

This is the first study, to the knowledge of the researcher, to document and describe unemployment rates in audiology graduates in South Africa. A similar study was done recently to describe human resource challenges in environmental health graduates in South Africa (Mbola, Human, & Melariri, 2019). Key findings from Mbola et al. (2019) did not include unemployment rates, but documented major problems regarding the decreasing number of employment opportunities for environmental health graduates (Mbola et al., 2019). Other associated reports of unemployment of healthcare professionals as a whole have mainly been in various media outlets over the last five years in South Africa with headlines such as ‘SA can’t give hundreds of its new doctors jobs’ (Ntsaluba, 2019).

### Community service program

It is important to note that in the first- and second-year postgraduation, since 1998, all South African health professional graduates are mandated to work for the National Department of Health (NDoH) for community service (CS) in order to be certified by the Health Professions Council of South Africa (HPCSA) as qualified-independent practitioners (Mbola et al., 2019; Reid, Peacocke, Kornick, & Wolvaardt, 2018). The intentions of the NDoH CS program were to combat the disparaging effects of the pre-1994 apartheid South African healthcare system that did not offer equal access to basic healthcare outside metropolitan areas (Reid et al., 2018). Therefore, a 16% – 19% unemployment rate for audiologists or any other health profession indicates that NDoH is failing to employ health professionals in line with their mandatory CS program. The South African public healthcare system has been cited to be facing, amongst other challenges, constrained financial resources that have led to difficulties in absorbing, retaining and remunerating all health professionals in the public healthcare system (Delobelle, 2013). It is also worth noting that the overall healthcare industry may also be influenced by the vast inequalities in the macro socio-economic sphere and the present COVID-19 factors that are not unique to healthcare but are seen to be impacting in all sectors in South Africa. Furthermore, recent research indicates that the South African economy as a whole is not growing fast enough to absorb the rising annual number of jobseekers, skilled such as health professionals or otherwise (Mahadea & Kaseeram, 2018).

This state of affairs has debilitating implications for the public healthcare system as well as the CS program and pertinent to this study, CS for audiologists. Therefore, it is key to consider that moving forward, if the NDoH is unable to sustainably maintain and fund CS, it is fitting to interrogate (1) the mandatory nature of the CS program as it stands for South African health graduates and (2) the linking of CS program to the health regulator’s, HPCSA, independent practice certification. Although well-intentioned, the mandatory CS program, as shown by this study findings, may be doubly disadvantaging health professional graduates as it not only cannot employ all graduates but further denies the unemployed graduates an opportunity to obtain the necessary accreditation to enter and serve in the broader healthcare market outside the NDoH (Mbola et al., 2019). Having any unemployed health professionals highlights a domino effect of negative ramifications that amount to a high loss of investment across multiple sectors (Pillay et al., 2020). Some of the key affected sectors include (1) higher education considering the cost attached to producing scarce skills, (2) the department of health with ‘wastage in the health workforce’ through unutilised critical skills and goals of healthcare access not being realised 20 + years post-apartheid for those in need and (3) the economy with non-contributing skilled labour adding to the already high (65%) unemployed youth under 35 years of age (Pillay et al., 2020). Post-COVID-19, there may be a place for strongly considering the incorporation of telehealth innovations not only for ensuring the much-needed hearing healthcare access but also to open up discussions on how to utilise technology to increase employment and business capacity amongst newly qualified health graduates.

### Education and skills do not equal employability?

In the last 20 years in post-apartheid South Africa, research proposed that increasing access, quality and levels of education will increase employability and benefit multiple sectors through skilled labour (Acquah, 2009; Lannoy et al., 2018; Mlatsheni & Rospabe, 2002). And through policy and action, the National Skills Development Strategy in line with the Skills Development Act of 1998 and Skills Development Levies Act of 1999 has seen South African tax revenue contribute financially to the goal of transforming access to and resourcing quality education and training (Pauw et al., 2006). However, the study findings may be indicating that the relationship between education and employability is not necessarily linear. For health professional graduates specifically, there may need to be a thorough investigation and understanding of the healthcare labour market’s capacity to absorb new graduates whilst retaining the current workforce and growing in line with the healthcare consumer needs (Mbola et al., 2019; Pillay et al., 2020). In particular to specialised fields like audiology, there may be a need for interrogation into the unique influences in the hearing healthcare market that in turn affect employability. The findings of this study are key for the hearing healthcare sector to consider career pathing for health professionals like audiologists in terms of considering alternative employment opportunities and options outside of the purely clinical sectors such as the NDoH.

### Telehealth and the future of health human resources

Globally, there is a growing interest and proven value in using technology in hearing healthcare (Aggarwal, Dhanshree Gunjawate, Yerraguntla, & Ravi, 2022; Eikelboom et al., 2022; Manchaiah et al., 2022). For South African audiology, telehealth may be a viable option to investigate how skills matching and human resource gaps can be facilitated to curb unemployment of hearing health professionals. Eikelboom et al. (2022) in their study assert that there is now more than ever within the COVID-19 climate, a positive outlook and potential for an increase in telehealth usage in audiology practice. Aggarwal et al. (2022) in their scoping review of the impact of COVID-19 on audiology practice also argue for careful considerations in planning for audiology practice post-pandemic. The findings of this study also imply that a thorough look into the future of audiology practice and what modes of employment can be sustainable to avoid health human resource wastage, and making audiology work during and post-COVID-19 is needed. Swanepoel and Hall (2020) put it simply that whilst COVID-19 is posing a tremendous threat, it is simultaneously inciting a rapid deployment of innovative telehealth approaches to respond to a changing landscape.

### Study strengths and limitations

This study is, to the knowledge of the researcher, the first to explore and document unemployment rates and challenges in audiologists in South Africa. This study’s findings can be foundational to guide future research to expand investigations into the subject matter. A possible limitation of the study is in its cross-sectional design in that findings cannot be used to predict and/or analyse unemployment over a period, and the snapshot nature of the design means that findings may not be representative of the whole population of interest.

## Conclusion

Up to 16% of audiologists are unemployed in their first-year postgraduation, and this increases to 19% in the second-year postgraduation. This study’s findings are the first to document the unemployment rate of newly graduated hearing healthcare professionals in South Africa. These findings have the potential to influence the critical discourse on hearing healthcare human resource policies and planning, hearing healthcare labour market needs versus capacity, and hearing healthcare context and potential for growth in the South African context, especially post-COVID-19.
